# Conference Report: NORTH AMERICAN INTEGRATED SERVICES DIGITAL NETWORK (ISDN) USERS’ FORUM (NIUF) Gaithersburg, MD October 26–30, 1992

**DOI:** 10.6028/jres.098.022

**Published:** 1993

**Authors:** Elizabeth B. Lennon

**Affiliations:** Computer Systems Laboratory, National Institute of Standards and Technology, Gaithersburg, MD 20899-0001

## 1. Introduction

The Computer Systems Laboratory (CSL), National Institute of Standards and Technology, sponsored the sixteenth North American ISDN Users’ Forum (NIUF) at its Gaithersburg, Maryland site on October 26–30, 1992. About 250 users, implementors, and service providers of ISDN technology attended the meeting. CSL collaborated with industry in 1988 to establish the NIUF to ensure that emerging ISDN applications meet the needs of users. A Cooperative Research and Development Agreement (CRDA) with industry was established in 1991 to govern the management of the forum; the CRDA now has 36 signatories from industry and academia. CSL serves as chair of the forum and hosts the NIUF Secretariat. NIUFmembership is open to all interested users, product providers, and service providers; meetings are held three times a year at various locations throughout North America.

## 2. The ISDN Standards Process

International standards for ISDN support worldwide communications for the exchange of voice, data, and image information among users, independent of any manufacturer, service provider, or implementation technology. ISDN standards are developed by the International Telephone and Telegraph Consultative Committee (CCITT) and, for North America in particular, by the Exchange Carriers Standards Association (ECSA) accredited standards committee, T1, under the umbrella of the American National Standards Institute (ANSI).

ISDN standards provide a broad variety of options and parameters to meet many potential needs and applications. To ensure interoperability and terminal portability within the ISDN network and its attendant equipment, a uniform subset of options and parameters must be selected for implementation. Each application usually requires only a subset of total functionality available in the standards; for ISDN products and services to work together in a multi-vendor environment, common sets of options must be selected.

To cope with this proliferation of choices and to provide practical products and services which meet the needs of users, the standards specification process must be extended to include the development of Application Profiles, Implementation Agreements, and Conformance Criteria which will promote interoperability. The NIUF addresses all of these areas.

## 3. NIUF Goals and Organization

The NIUF seeks to achieve three principal objectives:

- To promote an ISDN forum committed to providing users the opportunity to influence developing ISDN technology to reflect their needs;
- To identify ISDN applications, develop implementation requirements, and facilitate their timely, harmonized, and interoperable introduction; and
- To solicit user, product provider, and service provider participation in the process.

The actual work of the NIUF is accomplished in two workshops: the ISDN User’s Workshop (IUW) and the ISDN Implementor’s Workshop (IIW). The IUW produces Application Requirements which describe potential applications of ISDN and the features which may be needed. The IIW develops Application Profiles, Implementation Agreements, and Conformance Criteria which provide the detailed technical decisions necessary to implement an application requirement in an interoperable manner. The NIUF Executive Steering Committee coordinates the activities of the two workshops.

## 4. Accomplishments of the NIUF

Since its inception in 1988, the NIUF has achieved the following:

- 133 active applications for development of Application Profiles have been accepted;
- Application Profiles have been completed for 14 applications;
- 12 Implementation Agreements have been completed; and
- 8 Conformance Tests have been completed.

CSL established the NIST Special Publication 823 series, Integrated Services Digital Network Technology Publications, to publish the approved Implementation Agreements, Conformance Tests, and other NIUF documents. Copies of these documents are available for sale by the Government Printing Office, (202) 783-3238 or the National Technical Information Service, (703) 487-4650.

## 5. October 1992 NIUF Focuses on TRIP ‘92

The October NIUF focused on finalizing plans for the Transcontinental ISDN Project 1992 (TRIP ‘92) on November 16–20, 1992, which marked the advent of National Integrated Services Digital Network (National ISDN-1). Cosponsored by the NIUF and the Corporation for Open Systems (COS), TRIP ‘92 involved 74 user organizations in 149 locations across North America as well as several international sites. National ISDN-1 is based on national and international standards and is consistent with Implementation Agreements developed by the NIUF.

TRIP ‘92 Open Houses across the nation featured demonstrations of ISDN applications available to government and industry such as video/multimedia conferencing, desktop conferencing and screen sharing, LAN-to-LAN bridging, medical imaging, Group 4 facsimile, and image and large data file transfer. NIUF industry partners participating in the demonstrations at the NIST Open House site included AT&T Bell Laboratories, COMSAT, Bell Atlantic, and Northern Telecom. Also featured was a special session for about 170 government and industry executives. Speakers at this session included Robert M. White, Under Secretary for Technology, who presented a government view of national ISDN, and Irwin Dorros, Executive Vice President, Bellcore, who gave the industry perspective.

## 6. Highlights of October 1992 NIUF

Other highlights of the October meeting included a demonstration of Remote Message Retrieval Over ISDN by the Messaging and Answering Group and completion, subject to final approval at the NIUF February 1993 meeting, of two significant NIUF documents: *A Catalog of National ISDN Solutions for Selected NIUF Applications* and *A Generic Model for ISDN Cost Analysis.* Both documents resulted from a cross-industry team effort by NIUF members. The catalog focuses on demonstrating solutions for those applications identified as most important by an NIUF survey; most applications are satisfied with basic rate ISDN access and can be implemented using NI-1 solutions. The cost analysis document contains an ISDN checklist of both hard and soft costs and benefits. The document also includes a spreadsheet available in Microsoft Windows and MAC-based Excel, from which users can form the basis for their own customized spreadsheet for an ISDN cost analysis.

## 7. For More Information

For more information about the NIUF and its publications or to obtain conference proceedings, contact the NIUF Secretariat: Dawn Hoffman, Computer Systems Laboratory, National Institute of Standards and Technology, Building 223, Room B364, Gaithersburg, MD 20899-0001; telephone (301) 975-2937 or Fax (301) 926-9675.

